# The occurrence of and risk factors for depressive symptomatology in myocarditis survivors: a cross-sectional survey-based study using machine learning

**DOI:** 10.3389/fpsyt.2025.1581314

**Published:** 2025-04-28

**Authors:** Jean Marrero-Polanco, Laura Suarez Pardo, Shehzad K. Niazi, Daniel G. Smith, Cynthia J. Stoppel, Candace Moose, Arjun P. Athreya, Leslie T. Cooper, William V. Bobo

**Affiliations:** ^1^ Department of Molecular Pharmacology and Experimental Therapeutics, Mayo Clinic, Rochester, MN, United States; ^2^ Department of Psychiatry and Psychology, Mayo Clinic, Rochester, MN, United States; ^3^ Department of Psychiatry and Psychology, Mayo Clinic, Jacksonville, FL, United States; ^4^ The Myocarditis Foundation, Kingwood, TX, United States; ^5^ Department of Cardiovascular Medicine, Mayo Clinic, Jacksonville, FL, United States; ^6^ Department of Behavioral Science and Social Medicine, Florida State University College of Medicine, Tallahassee, FL, United States

**Keywords:** depression, anxiety, traumatic distress, psychosocial factors, myocarditis

## Abstract

**Background:**

The frequency and impact of depressive symptoms in myocarditis survivors are poorly understood.

**Objectives:**

We conducted a cross-sectional study to identify risk factors and the relative importance of each for predicting clinically significant depressive symptomatology in myocarditis survivors.

**Methods:**

Participants completed an electronic survey assessing sociodemographic, general health, and myocarditis-related variables, as well as self-reported cardiac symptoms and personal and family mental health history. Participants also completed the Center for Epidemiologic Studies Depression Scale (CES-D), Beck Anxiety Inventory (BAI), revised Impact of Events Scale (IES-R), and other validated measures of social support, quality of life, resiliency, childhood adversity, treatment distress, and somatic symptom burden. Clinically significant depressive symptomatology was defined as a CES-D total score ≥ 16. We used supervised machine learning to examine which and how well psychosocial and other types of variables predicted clinically significant depressive symptomatology in myocarditis survivors. Finally, we calculated the variable importance for each variable from the trained models and examined the rank ordering of predictors.

**Results:**

Ninety-six of 113 respondents (85.0%) with complete survey data were included in the analyses. Forty-three (44.8%) respondents had clinically significant depressive symptomatology. When predicting depressive symptomatology, random forests achieved a mean AUC of 0.91 (95% CI 0.87-0.95) and a significantly higher accuracy than that of the null information rate (0.84 vs 0.55, *p* < 0.005), with correspondingly high sensitivity (0.84) and specificity (0.85). Emotional wellbeing, quality of life, history of depression, anxiety, and resilience were the top predictors in variable importance analyses, ahead of self-reported cardiovascular symptoms, other myocarditis-related variables, and family history of depression.

**Conclusions:**

Myocarditis survivors are at high risk for clinically significant depressive symptomatology. Psychosocial factors that are measurable in routine practice may be more predictive of significant depressive symptomatology than demographics, family history, or self-reported cardiovascular symptoms.

## Introduction

Myocarditis is an inflammatory disease of the myocardium resulting in ventricular systolic dysfunction (1). Myocarditis accounts for up to 10% of acute-onset heart failure cases and for a substantial proportion of life-threatening dysrhythmias (2, 3). Indeed, myocarditis presents acutely along a spectrum of severity ranging from asymptomatic cases to fulminant illness, the latter associated with severe myocardial injury and events of cardiogenic shock and sudden cardiac arrest (4, 5).

Depression affects over 350 million people worldwide and is a leading cause of chronic disability (6). Depression is associated with poor functioning across life domains (7) and premature mortality due to suicide and general medical causes (8, 9). The prevalence of depression is particularly high in people living with cardiovascular diseases, and there are known associations of prevalent depression with incident coronary artery disease (CAD) and heart failure (HF) and vice-versa, suggesting temporally bi-directional relationships between depression and these cardiac conditions (10–12). In these patients, the presence and severity of depression are linked to worse clinical outcomes, low quality of life, increased physical disability, and high mortality (13).

Despite foreseeable risks (14), research on the occurrence of depressive symptomatology in myocarditis survivors is limited. One cross-sectional study of 41 patients hospitalized for acute myocarditis found depressive and posttraumatic stress symptoms in 27% of cohort members (15). In a separate follow-up study, 46% of 249 adolescents/young adults who developed myocarditis after mRNA COVID-19 vaccination reported problems with depression or anxiety (16). While these studies documented high prevalences of depressive symptoms similar to those observed in patients with CAD and HF (17, 18), both were limited by short follow-up and a lack of information on risk factors for depression.

Studying the occurrence of and risk factors for depressive symptomatology in myocarditis survivors is crucial, considering the distress associated with sudden and potentially life-threatening cardiac illness (19). Although the prognosis for surviving acute myocarditis is favorable, many patients struggle with chronic HF, persisting cardiac symptoms, or sudden recurrent symptoms (20). Factors affecting long-term functioning and quality of life during survivorship, such as depression, may be as important as acute predictors of mortality.

This study aimed to describe the prevalence of clinically significant depressive symptomatology in a sample of myocarditis survivors and identify potential predictors of depression within the cohort. A secondary objective was to derive a quantitative (statistical) rank-ordering of the relative importance of individual variables (including sociodemographic, clinical, family history, and psychosocial variables) for each prediction model using relative importance analysis. We hypothesized that psychosocial variables would be more predictive of depressive symptomatology than self-reported cardiac symptom burden, demographic variables, and family history of depression in myocarditis survivors.

## Materials and methods

### Study design

The Mayo Clinic Institutional Review Board approved the study protocol. We conducted a cross-sectional, survey-based study to identify risk factors for clinically significant depressive symptomatology and to assess the relative importance of a variety of psychosocial and other cardiac, behavioral, and sociodemographic variables in a cohort of myocarditis survivors using logistic regression and machine learning (**Supplementary Figure 1**).

### Subjects and recruitment

We recruited adults (≥ 18 years old), with a self-identified history of myocarditis (including fully recovered individuals and those with active illness or cardiac symptoms) via electronic messages, internet advertising at Mayo Clinic and the Myocarditis Foundation, and by referral from primary care and cardiovascular medicine clinics at Mayo Clinic. We excluded individuals with evidence of decompensating HF (dyspnea, lower extremity swelling, or fatigue requiring hospitalization). Participants had to understand written English. Informed consent was obtained electronically or by written signature. Only surveys with valid informed consent were included.

### Survey elements

A comprehensive survey was developed to measure depressive symptoms, gather relevant myocarditis-specific information, and assess other factors that may link depression and severe cardiac disease based on previous research. Given a central mediating role of chronic stress for both depression and cardiovascular dysregulation (21), we were interested in assessing important stress correlates (anxiety symptoms, health anxiety, traumatic stress, quality of life), moderating factors (social support, resiliency, childhood adversity), and external causes (treatment distress, somatic and cardiac symptom burden). Eligible participants completed an electronic survey, launched using Qualitrics with back-end data management via RedCap, that assessed sociodemographic characteristics, spirituality, general health variables, myocarditis-related variables, caregiving responsibilities, care-receiving status, and personal and family mental health history (**Supplementary Table 1**). The time frame for participant data collection occurred between July 2021 and June 2023. We assessed the following five additional domains using validated assessment instruments.

- Depressive symptomatology:Depressive symptoms were assessed using the Center for Epidemiologic Studies Depression Scale (CES-D); clinically significant depressive symptomatology was defined as a CES-D total score ≥ 16 (22, 23).
- General anxiety, traumatic distress, and health anxiety: Clinically significant general anxiety and traumatic distress were defined based on validated cut-off scores of 22 and 24 on the Beck Anxiety Inventory (BAI) and the revised Impact of Events Scale (IES-R), respectively (24–26). Health anxiety was assessed using the short version of the Health Anxiety Inventory (HAI) (27).
- Quality of life, social support, resiliency, and childhood adversity: Quality of life and wellbeing, social support, resilience to stress, and childhood adversity were measured using the Linear Analog Self-Assessment (LASA) (28, 29), the ENRICHD Social Support Instrument (ESSI) (30), the Brief Resilience Scale (BRS) (31), and the Adverse Childhood Experiences (ACE) questionnaire (32), respectively.
- Treatment distress: We assessed sources of distress related to treatment and interacting with the medical system using an adaptation of the Cancer and Treatment Distress Scale (CTXD) (33). Although the CTXD was validated in oncology, specific items apply to diseases like myocarditis. Therefore, to ensure relevance to myocarditis survivors, 5 cancer-specific items were dropped from the original CTXD, leaving 17 modified items (each rating levels of distress or worry on a 0 [none] to 3 [severe] point scale) whose sum of scores generated a “modified CTXD” score. The modified CTXD items assessed perceived levels of distress or worry related to factors of relevance to myocarditis survivors including activity restriction, relying on others, long-term treatment effects, costs of care, dealing with the medical system and health insurance, communicating with healthcare workers, emotional toll on significant others, changes in appearance, things going wrong, inability to care for family, returning to work, and feeling like a burden to others.
- Somatic and cardiac symptoms: The 15-item Public Health Questionnaire (PHQ-15) assessed general somatic symptom burden (34). Burden from cardiac symptoms was estimated by summing the scores of PHQ-15 items 6 (chest pain), 7 (dizziness), 8 (faintness), 9 (palpitations), 10 (shortness of breath), and 14 (fatigue). Although these symptoms are somewhat ubiquitous, myocarditis survival may increase their odds of being cardiac-related. For these six symptoms, respondents were asked to rate perceived changes in severity over the prior six months (or since diagnosis if <6 months) on the following scale: 0 (no change), 1 (a little worse), and 2 (much worse).

A paper version of the survey was available to participants upon request. The electronic and paper surveys included prompts to take periodic breaks and messages of encouragement to optimize motivation.

### Descriptive statistics

Demographic and clinical variables were summarized using proportions for categorical values and medians (with interquartile ranges) for continuous values. We compared proportions using Fisher exact tests and continuous values using Wilcoxon rank-sum tests with continuity correction, exact Wilcoxon rank-sum test (in cases of statistical ties), or two-sample t-test (adjusted for unequal variances).

### Machine learning workflow

Supervised machine learning (ML) methods were used to identify independent sociodemographic, clinical, and behavioral/psychosocial predictors of clinically significant depressive symptomatology. Multiple logistic regression (adjusting for age, sex, smoking status, alcohol intake, and history of depression before enrollment) was used as an additional test of independence for psychosocial measures showing significant differences between depressed and non-depressed respondents and for the top five predictors from the ML models.

### Prediction models

Random forest, extreme gradient-boosted decision tree-based learning (XGBoost), and penalized regression (Pen-Reg) methods were examined as prediction performance of methods are not known *a priori* due to potential non-linear relationships in the data associating with predicted outcomes. Given these three methods have varying degrees of evidence in predicting depressive phenotypes (35, 36), this work chose to experiment a collection of methods that are classification algorithms with slightly different approaches, albeit with a common goal of deriving optimal predictive capabilities. First, the random forest model aggregates predictions from multiple decision trees that use random subsets of the data, a technique called bagging (37). Second, the XGBoost model was also built using multiple decision trees; however, rather than only aggregating predictions, it sequentially builds decision trees, where each tree corrects errors from the previous one through gradient boosting, resulting in a stronger predictive model (38). Finally, the Pen-Reg model was built by using the glmnet method which utilizes lasso, ridge, or elastic-net regularization (determined in model tuning) to shrink or eliminate non-informative variables from the prediction model (39, 40). A comprehensive list of features (predictor variables) used in the models for classifying clinically significant depressive symptomatology is presented in **Supplementary Table 2**.

### Data Preprocessing and Model Development

Pre-processing began by excluding variables with missingness exceeding 25%. For the remaining missingness, k-nearest neighbor imputation was used. Variables with near-zero variances were excluded, determined by default tuning parameters (frequency distribution ratio=95/5 and percent of unique values=10). Observations were excluded from the analysis if any CES-D item was left unanswered. Nested cross-validation was used to train the prediction models for clinically significant depressive symptomatology. For ten iterations of the outer loop of the nested resampling, the data was randomly split into 70/30 train/test. Given the percentage of myocarditis survivors with clinically significant depressive symptomatology (44.8%), upsampling was used to minimize class imbalance. Within the inner loop of the nested resampling, 10-fold cross-validation with three repeats was performed on the training set for hyperparameter tuning.

### Model Performance

Each model’s prediction performance was evaluated using the mean area under the receiver operating characteristic curve (AUC), where a value of 0.50 indicates random guessing and a value of 1.0 indicates perfect predictions. The significance of prediction performance was assessed by comparing the model’s accuracy to the null information rate (NIR, defined as the larger proportion of the predicted outcomes), which served as a proxy for chance. The mean and the 95% confidence interval (CI) for each metric (AUC, accuracy, sensitivity, specificity, positive predictive value [PPV], and negative predictive value [NPV]) were calculated based on the results obtained from the ten outer iterations.

### Variable Importance

After training the models, variable importance (VI) scores for model variables predicting clinically significant depressive symptomatology was assessed using the permutation method. The importance of a variable in the prediction model relative to all remaining variables was measured by permutating (randomly shuffling) the values of a variable and observing the impact of the model’s performance (41). If in the absence of a variable the model’s performance decreases, measured by an increase in error, it indicates the importance of the variable. The VI scores were standardized from 0 to 100.

### Data and Code Availability

There analyses were conducted in R v4.2.2 using RStudio version 1.3 (42, 43). Model training and testing were performed using the tidymodels package version 0.1.2. All programming scripts and data are available upon reasonable request to the corresponding author.

## Results

### Demography and clinical characteristics

Of the 113 returned surveys, 96 had complete CES-D responses and were included in the analysis. As shown in **Table 1**, respondents were predominantly Caucasian, unmarried, employed, and educated persons residing in North America, with a relatively even split between women and men. The median time interval between myocarditis diagnosis and survey completion was four years. The most common self-reported types of myocarditis were viral (41.7%) and giant cell myocarditis (12.5%), while 20.8% of respondents reported an unknown cause (**Table 2**). Of the 96 respondents, 19 (19.8%) reported using an automated implantable cardioverter defibrillator (AICD), 11 (11.5%) reported needing a device to maintain cardiac functioning, and 11 (11.5%) reported receiving a myocardial transplant.

**Table 1:** Demographic and clinical characteristics of the survey respondents, overall and by clinically significant depressive symptomatology status^a^.

	Overall cohort, n=96	Clinically significant depressive symptomatology, n=43	Non-depressed, n=53	p-value^b^
	Median (IQR)	Median (IQR)	Median (IQR)	
Age, years	41.0 (31.0, 56.0)	35.5 (26.2, 45.0)	50.0 (38.0, 60.5)	0.001
Age at myocarditis diagnosis, yrs	35.0 (25.0, 51.0)	32.0 (22.0, 40.5)	41.0 (30.0, 54.0)	0.004
	n (%)	n (%)	n (%)	p-value^b^
Female sex	49 (51.0)	22 (51.2)	27 (50.9)	1.000
Race/ethnicity				0.760
Caucasian	69 (71.9)	30 (69.8)	39 (73.6)	
African American	6 (6.3)	3 (7.0)	3 (5.7)	
Hispanic	2 (2.1)	0	2 (3.8)	
East Asian	1 (1.0)	1 (2.3)	0	
South Asian	3 (3.1)	2 (4.7)	1 (1.9)	
Middle Eastern	6 (6.3)	2 (4.7)	4 (7.5)	
No response	9 (9.4)	5 (11.6)	4 (7.5)	
Region of residence				0.170
North America	82 (85.4)	34 (79.1)	48 (90.6)	
South America	1 (1.0)	1 (2.3)	0	
Europe	7 (7.3)	5 (11.6)	2 (3.8)	
Africa	1 (1.0)	0	1 (1.9)	
Asia	2 (2.1)	2 (4.7)	0	
Australia	3 (3.1)	1 (2.3)	2 (3.8)	
Marital status				0.080
Married or partnered	23 (24.0)	14 (32.6)	9 (17.0)	
Unmarried	69 (71.9)	26 (60.5)	43 (81.1)	
No response	4 (4.2)	3 (7.0)	1 (1.9)	
Highest level of education				0.540
Some college	73 (76.0)	33 (76.7)	40 (75.5)	
High school	17 (17.7)	6 (14.0)	11 (20.8)	
Less than high school	5 (5.2)	3 (7.0)	2 (3.8)	
No response	1 (1.0)	1 (2.3)	0	
Employment status				0.005
Full-time employed	51 (53.1)	32 (74.4)	19 (35.8)	
Part time employed	14 (14.6)	4 (9.3)	10 (18.9)	
Unemployed	21 (21.9)	5 (11.6)	16 (30.1)	
Other	6 (6.3)	1 (2.3)	5 (9.4)	
Disabled, cannot work	3 (3.1)	1 (2.3)	2 (3.8)	
No response	1 (1.0)	0	1 (1.9)	
Household income, annual, USD				1.000
$75,000 - $100,000 USD	62 (64.6)	28 (65.1)	34 (64.2)	
Less than $75,000	24 (25.0)	11 (25.6)	13 (24.5)	
No response	10 (10.4)	4 (9.3)	6 (11.3)	

a Clinically significant depressive symptomatology was defined as a CES-D total score >16.

b Comparisons between depressed and non-depressed respondents used Wilcoxon rank-sum (with continuity correction) or Fisher exact tests.

**Table 2:** Clinical characteristics of survey respondents.

	Overall cohort, n=96	Clinically significant depressive symptomatology, n=43	Non-depressed, n=53	p-value
	n (%)	n (%)	n (%)	
Clinically significant anxiety Missing/unknown	21 (21.9) 11 (11.5)	17 (39.5) 4 (9.3)	4 (7.5) 7 (13.2)	<0.001
Significant traumatic distress Missing/unknown	33 (34.4) 8 (8.3)	26 (60.5) 3 (7.0)	7 (13.2) 5 (9.4)	<0.001
Smoking status				0.014
Never smoker Former smoker Active smoker	82 (85.4) 12 (12.5) 2 (2.1)	32 (74.4) 9 (20.9) 2 (4.7)	50 (94.3) 3 (5.7) 0	
Alcohol use in the past year				0.025
Not at all Less than once weekly Less than half of days/week Most days of the week Daily	25 (26.0) 40 (41.7) 26 (27.1) 3 (3.1) 2 (2.1)	8 (18.6) 24 (55.8) 8 (18.6) 1 (2.3) 2 (4.7)	17 (32.1) 16 (30.2) 18 (34.0) 2 (3.8) 0	
Recreational drug use				0.005
Never used Former use (not active) Occasional use Daily use	71 (74.0) 21 (21.9) 3 (3.1) 1 (1.0)	25 (58.1) 15 (34.9) 2 (4.7) 1 (2.3)	46 (87.0) 6 (11.3) 1 (1.9) 0	
Weekly exercise/activity level				0.053
None One to three days Most days or every day	13 (13.5) 39 (40.6) 44 (45.8)	8 (18.6) 21 (48.8) 14 (32.6)	5 (9.4) 18 (34.0) 30 (56.6)	
Cause of myocarditis				0.057
Viral Bacterial Giant cell myocarditis Immune disease Medication allergy Unknown cause Missing/unknown	40 (41.7) 3 (3.1) 12 (12.5) 8 (8.3) 7 (7.3) 20 (20.8) 6 (6.3)	21 (48.8) 1 (2.3) 2 (4.7) 4 (9.3) 6 (14.0) 8 (18.6) 1 (2.3)	19 (35.8) 2 (3.8) 10 (18.9) 4 (7.5) 1 (1.9) 12 (22.6) 5 (9.4)	
Hospitalization for myocarditis				0.726
None Once 2-4 times 5-7 times 8-10 times >10 times	45 (46.9) 27 (28.1) 18 (18.9) 1 (1.0) 1 (1.0) 4 (4.2)	19 (44.2) 12 (27.9) 9 (20.9) 0 0 3 (7.0)	26 (49.1) 15 (28.3) 9 (17.0) 1 (1.9) 1 (1.9) 1 (1.9)	
AICD^a^ use Missing/unknown	19 (19.8) 2 (2.1)	6 (14.0) 1 (2.3)	13 (24.5) 1 (1.9)	0.484
Device needed to maintain cardiac functioning^b^ Missing/unknown	11 (11.5) 2 (2.1)	3 (7.0) 1 (2.3)	8 (15.1) 1 (1.9)	0.529
Received myocardial transplant Missing/unknown	11 (11.5) 2 (2.1)	4 (9.3) 1 (2.3)	7 (13.2) 1 (1.9)	0.875
Amongst myocarditis survivors with depression history, n=50				
	Depression treatment			p-value
		Clinically significant depressive symptomatology, n=32	Non-depressed, n=18	1.000
		n (%)	n (%)	
	Yes	24 (75.0)	13 (72.0)	
	No	8 (25.0)	5 (28.0)	
	Depression relative to myocarditis			0.002
	Depression only after	5 (16.0)	7 (39.0)	
	Depression before and after	24 (75.0)	5 (28.0)	
	Depression only before	2 (6.0)	6 (33.0)	
	Missing/unknown	1 (3.0)	0 (0.0)	
	Perceived depression linked with myocarditis			0.451
	Entirely (or almost entirely) related	6 (19.0)	4 (22.0)	
	Mostly related	10 (31%)	2 (11.0)	
	Slightly related	8 (25.0)	5 (28.0)	
	Not related	8 (25.0)	7 (39.0)	

a AICD, automatic implantable cardioverter defibrillator.

b Including use of a ventricular assist device, intra-aortic balloon pump, or extracorporeal membrane oxygenation.

### Characteristics of myocarditis survivors with clinically significant depressive symptomatology

A total of 43 (44.8%) respondents were classified as having clinically significant depressive symptomatology based on a CES-D total score ≥ 16. Depressed respondents were significantly younger during survey completion and at myocarditis diagnosis than non-depressed respondents (**Table 1**) and had higher rates of smoking, alcohol consumption, histories of recreational drug use, and diagnosed depression before study enrollment (**Table 2**). Nine (20.9%) of 43 respondents with CES-D total scores ≥ 16 had no current or past depression diagnoses. Among the 50 cohort members with a history of diagnosed depression, 13 (26.0%) reported having received no treatment, and 22 (44.0%) perceived that their depression was entirely or mostly related to their myocarditis diagnosis or symptoms (**Table 2**).

**Figure 1:**
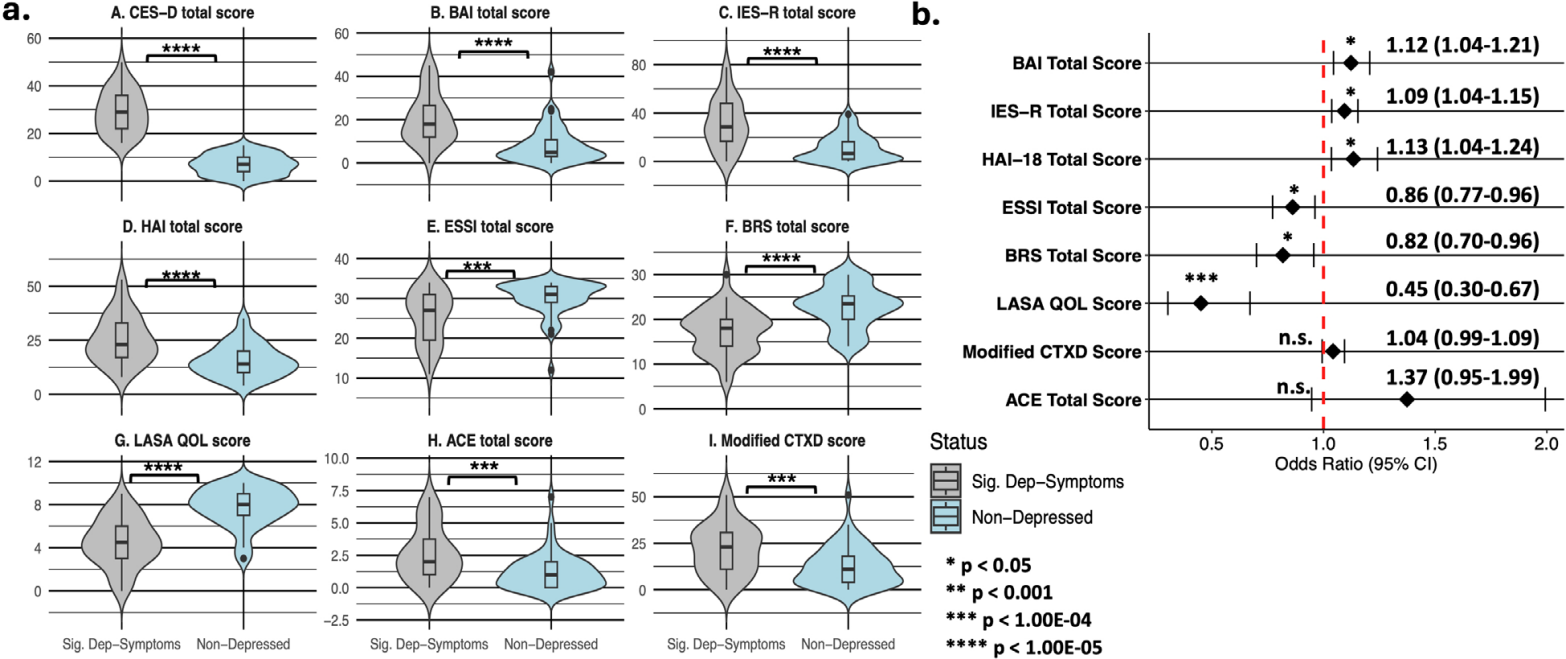
Psychosocial measure distributions stratified by clinically significant depressive symptomatology status **(a)** and adjusted logistic regression analysis **(b)** in 93 myocarditis survivors. Panel A summarizes comparisons of median (IQR) values for symptoms of depression (panel A), general anxiety (panel traumatic distress (panel C), health anxiety (panel D), social support (panel E), resiliency (panel F), quality of life (panel G), childhood adversity (panel H), and treatment distress (panel I) between respondents with clinically significant depressive symptomatology (CES-D total score ≥ 16) and non-depressed survey respondents. Panel B shows the results of multiple logistic regression models (adjusting for age at survey completion, sex assigned at birth, smoking status, alcohol intake, and self-reported history of depression prior to survey initiation) that test the association of clinically significant depressive symptomatology with measures from panels B-I in **Figure 1a**. Abbreviations are defined as follows: ACE, Adverse Childhood Experiences questionnaire; BAI, Beck Anxiety Inventory; BRS, Brief Resiliency Scale; CES-D, Center for Epidemiologic Studies Depression scale; ESSI, ENRICHD Social Support Instrument; HAI, Health Anxiety Inventory (short form); IES-R, revised Impact of Events Scale; LASA QOL, Linear Analog Self-Assessment (of overall and domain-specific quality of life); modified CTXD, modified version of the Cancer and Treatment Distress measure.

As compared to respondents without clinically significant depressive symptomatology, those with it had higher rates of clinically significant anxiety and traumatic distress, as well as higher median CES-D, BAI, IES-R, HAI, ACE questionnaire, and modified CTXD scores—and significantly lower ESSI and LASA (overall quality of life) scores (**Figure 1a**, **Supplementary Table 3**). The scores on each scale, except ACE and modified CTXD scores, were also significantly associated with depressive symptomatology in adjusted logistic regression models (**Figure 1b**). PHQ-15 total scores, sub-scores indicating the number of cardiac symptoms, and their respective severities were also significantly higher in depressed respondents (**Supplementary Table 3**).

### Prediction of clinically significant depressive symptomatology

**Figure 2:**
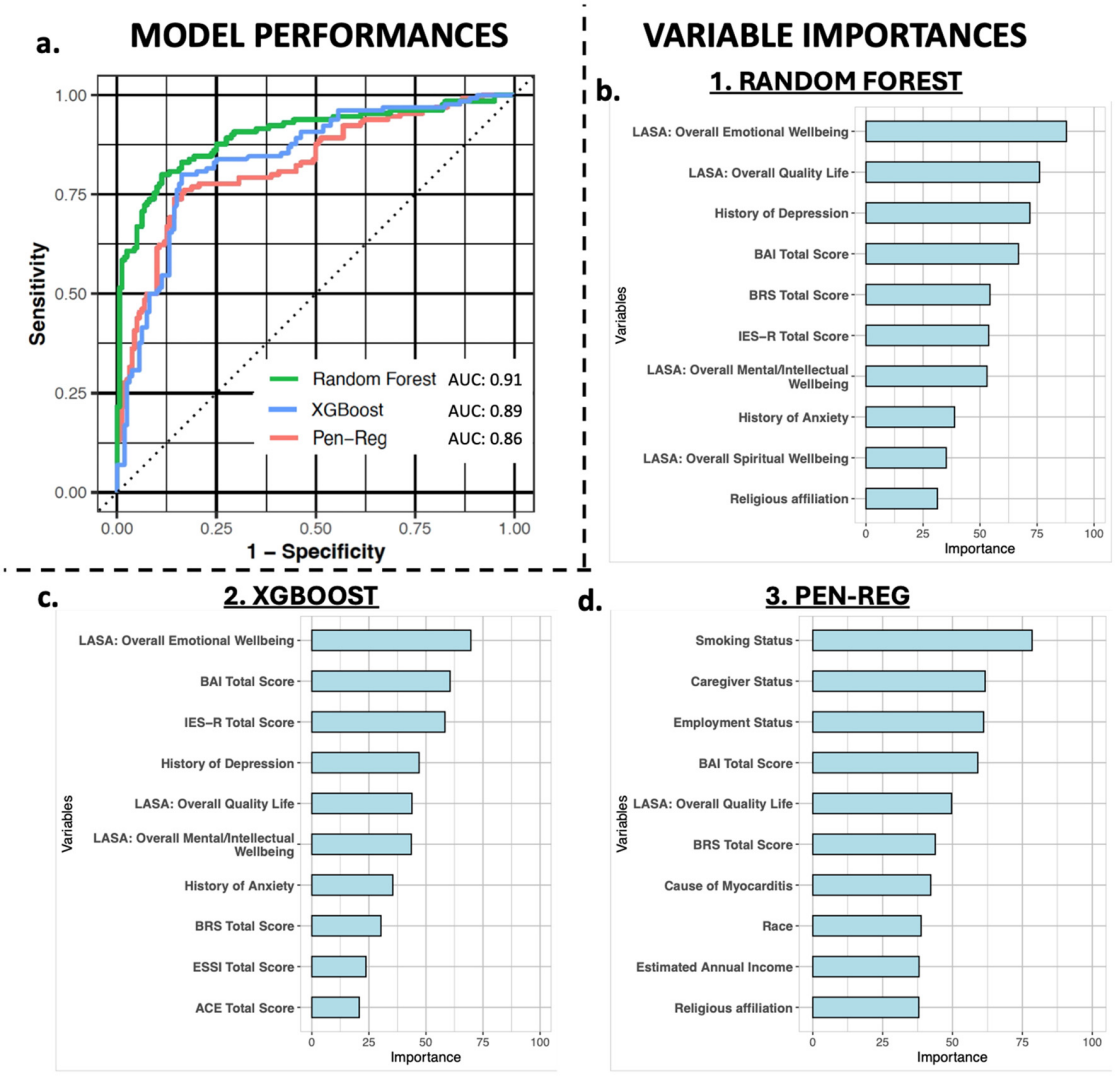
Model performance and variable importance. Panel **(a)** shows the AUCs of the random forest (green), XGBoost (blue), and penalized regression (Pen-Reg, pink) algorithms for predicting clinically significant depressive symptomatology. Panels **(b–d)** graphically display the variable importance metrics for the top 10 predictive variables in random forest, XGBoost, and Pen-Reg models, respectively.

Supervised machine learning methods were used to predict clinically significant depressive symptomatology. After cross-validation, the random forest model achieved the best-performing mean AUC of 0.91 (95% CI 0.87-0.95) and an accuracy that was significantly higher than the NIR (0.84 vs 0.55, *p* < 0.005), with balanced sensitivity and specificity, compared to XGBoost (AUC: 0.89) and Pen-Reg (AUC: 0.86) (**Table 3**, **Figure 2a**). During VI analysis, the top predictors of clinically significant depressive symptomatology from the random forest model included LASA emotional wellbeing and overall quality of life scores, history of depression before study enrollment, and total scores on the BAI, BRS, and IES-R (**Figure 2b**, **Supplementary Table 4**). XGBoost top predictors included the same variables as in random forest but in differing rank order (**Figure 2c**, **Supplementary Table 5**). Although Pen-Reg top predictors did include anxiety, quality of life, and resilience, other variables (smoking, employment, and caregiver statuses) were more predictive (**Figure 2d**, **Supplementary Table 6**). Adjusted logistic regression analyses on top predictors from the random forest and XGBoost models confirmed independent associations of clinically significant depressive symptomatology with LASA overall emotional wellbeing and quality of life scores, as well as total scores on the BRS, IES-R, and BAI (**Supplementary Table 7**).

**Table 3:** Prediction performance metrics for extreme gradient-boosted decision tree (XGBoost) models predicting clinically significant depressive symptomatology.

Clinically Significant Depressive Symptomatology Model			
Random Forest			
Metrics	Mean	95% CI	p-value^a^
Accuracy	0.84	0.80, 0.89	0.005
NIR^b^	0.55		
AUC	0.91	0.87, 0.95	
Sensitivity	0.84	0.76, 0.91	
Specificity	0.85	0.79, 0.91	
PPV	0.83	0.77, 0.89	
NPV	0.87	0.82, 0.93	
XGBoost			
Accuracy	0.78	0.73, 0.83	0.02
NIR^b^	0.55		
AUC	0.89	0.85, 0.93	
Sensitivity	0.79	0.71, 0.88	
Specificity	0.77	0.71, 0.83	
PPV	0.74	0.69, 0.79	
NPV	0.83	0.77, 0.89	
Penalized Regression			
Accuracy	0.78	0.78, 0.80	0.009
NIR^b^	0.55		
AUC	0.86	0.83, 0.89	
Sensitivity	0.73	0.64, 0.82	
Specificity	0.82	0.78, 0.86	
PPV	0.77	0.75, 0.80	
NPV	0.80	0.75, 0.85	

AUC, area under the receiver operating characteristic curve; NIR, null information rate; NPV, negative predictive value; PPV, positive predictive value.

a p-values are for comparisons of the accuracies of the predictive model vs the NIR.

b The NIR is a proxy for chance based on the current distribution of the label (depressive symptomatology).

## Discussion

Advances in the diagnosis and care of myocarditis have resulted in varying but generally favorable short-term prognoses for many patients (44). However, questions about long-term effects on mental health and other patient-centered outcomes in myocarditis survivors remain. This cross-sectional, survey-based study adds to the existing research on the mental health of patients with a history of myocarditis by documenting high rates of clinically significant depressive symptomatology (44.8%), as well as general anxiety (21.9%) and traumatic distress (34.4%), using validated assessments and cut-off scores.

As of this writing, there is limited evidence on the occurrence of neuropsychiatric symptoms in patients with myocarditis. In a study by Mirabel and colleagues (2011) (15), the prevalence of depression and anxiety, as measured by the Hospital Anxiety and Depression Scale (HADS) (45), was 27% and 38%, respectively—and 27% for posttraumatic distress, as measured by the IES-R—among 41 patients admitted to intensive care units with severe myocarditis requiring a VAD or ECMO. Psychiatric symptoms were apparent 525 days after discharge from the ICU despite improved cardiac functioning for survivors. Kracalik and colleagues (2022) documented a higher prevalence of depression (46%) (16), defined using the Euro-QoL 5-dimension, 5-level (EQ-5D-5L) questionnaire (46), in a cohort of 249 persons, aged 12-29 years, assessed 90 days after the onset of myocarditis following mRNA COVID-19 vaccination. As in the Mirabel et al. (2011) report (15), the prevalence of depression was high despite good cardiac recovery. In our study, rates of significant symptoms of depression, general anxiety, and traumatic distress were comparably high after a median of 4 years following myocarditis diagnosis, with potentially good cardiac recovery suggested by relatively high weekly exercise frequencies and low rates of disability or inability to work. Collectively, these findings point to a high risk of adverse mental health conditions in myocarditis survivors despite high cardiac recovery rates.

An elevated risk of new-onset or exacerbated depression in myocarditis survivors may be anticipated, given its known association with cardiac diseases (17). The life-threatening nature of myocarditis in its acute phases and possible persisting complications, recurrences, and disability may heighten the risk for depression (19, 47), highlighting the need for studies investigating risk factors of depression in myocarditis survivors. To our knowledge, this is the first study identifying factors of greater importance for predicting clinically significant depressive symptomatology in myocarditis survivors with a rank-ordering of their relative importance using supervised ML. Psychosocial factors were among the most important predictors, relative to the contributions from demographic and self-reported cardiac symptom variables in the best performing random forest model. Given the known contributions of psychosocial factors in depression and the likelihood of cardiac recovery for most of our cohort members (48), this finding is perhaps unsurprising. Nevertheless, the identified top 10 risk factors already have known associations with both depression and with heightened cardiac disease risk or worse cardiovascular prognosis (48–50), suggesting reasonable ecological validity.

Depression in myocarditis survivors is a complex phenotype with a wide network of interacting disease risk factors. Accordingly, the predictive variables in this study were numerous. They covered various constructs, including sociodemographic characteristics, detailed mental health and cardiovascular phenotyping characteristics, somatic symptoms, perceptions of health and spirituality, physical activity and sleep, substance exposures, and a focused set of psychosocial measures. Since the rate of depression in myocarditis survivors was not known *a priori*, the current study was not powered for a target prediction performance (e.g., an AUC of 0.80). Future work should evaluate and validate model performance and top predictors with independent datasets and from diverse populations. If validated, this work may serve as a template for future studies identifying highly predictive variables–or combinations of predictive variables–to characterize myocarditis survivors who may require closer follow-up based on post-acute risk for depression. This need is foreseeable given new and emerging causes of myocarditis and post-acute risks despite generally favorable odds of survivorship (51).

### Study limitations

We were unable to ascertain the timing of depressive symptoms relative to the onset of myocarditis. Although 44% of people with a history of depression believed that their depression was mostly or entirely related to myocarditis, over half reported having depression before and after myocarditis diagnosis, and less than a quarter reported depression only after myocarditis diagnosis. Due to the cross-sectional nature of this study, causal inference based on these findings is limited. Detailed longitudinal studies are needed to establish time-dependent associations of and risk factors for depression and anxiety in myocarditis survivors. Second, despite high predictive accuracies, the performance of the ML models was limited to input data. Several factors—both biological and contextual—were not measured. Third, although repeated cross-validation was used, there were no external samples for validation. Fourth, the study’s relatively small sample size prevented the identification of predictive factors for clinically significant depressive symptomatology within cause-specific subgroups of myocarditis survivors. Instead, data were analyzed in aggregate, regardless of myocarditis etiology. Fifth, survey methods in this study assessed neuropsychiatric symptoms using self-report instruments and validated cut-off scores without confirmation of psychiatric diagnoses. Sixth, our measures of subjective cardiac symptom burden were few, were relatively crude, and were applied long after index myocarditis episodes. We could not determine whether the relative importance of cardiac symptoms might have changed using direct cardiac performance measures or in more acutely ill or disabled cohorts. Finally, additional studies are needed in more racially and socioeconomically diverse samples given higher prevalences of cardiovascular risk factors and worse cardiovascular disease outcomes in minoritized patients and the impact of correlated disparities in healthcare access and social determinants of health (52, 53).

## Conclusions

Myocarditis survivors appear to be at high risk for clinically significant depressive symptomatology, general anxiety, and posttraumatic distress, consistent with prior research. We extend existing literature by identifying factors of greater importance for predicting clinically significant depressive symptomatology in myocarditis survivors using ML. Given the high rates of myocarditis survival, this work highlights the importance of modeling survivorship-centered mental health over time and the significance of assessing psychosocial factors for predicting risks.

## Data Availability

Requests for the data or code used in this study can be made to the Mayo Clinic Ventures and will be reviewed as per Mayo Clinic data management policies. Requests to access the datasets should be directed to WB, william.bobo@med.fsu.edu.
