# Supplementary material for: The occurrence of and risk factors for depressive symptomatology in myocarditis survivors: a cross-sectional survey-based study using machine learning
Source: Front Psychiatry. 2025 Apr 28;16:1581314. doi: 10.3389/fpsyt.2025.1581314 (PMC12066494; doi:10.3389/fpsyt.2025.1581314)
Supplement: Supplementary file 1 [file DataSheet1.pdf]

SUPPLEMENTARY FIGURE LEGEND

Supplementary Figure 1. Workflow overview. We used data from an electronic survey to examine predictors of clinically significant depressive symptomatology (based on a total score cut-off of 16 on the Center for Epidemiological Studies-Depression [CES-D] scale) in 96 myocarditis survivors with complete survey responses. Supervised machine learning, cross-validation, and variable importance analysis using complete survey data was conducted. Multiple logistic regression analyses were used to test the associations of top predictors identified from the variable importance analyses, adjusted for sex, age, smoking status, alcohol intake, and self-reported history of depression.

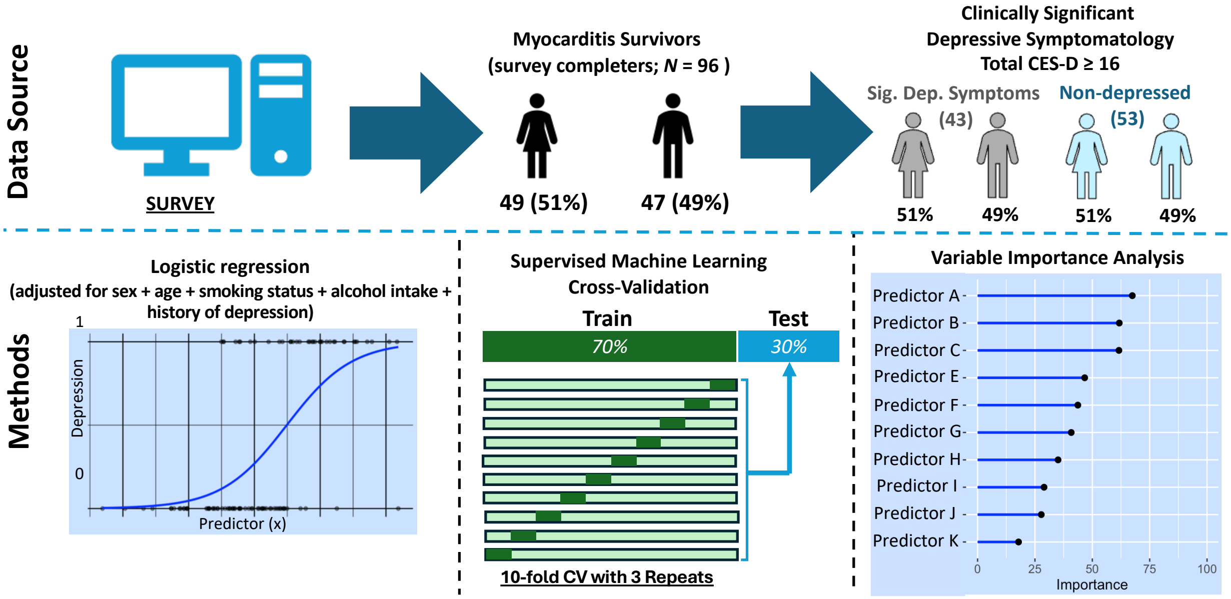

Supplementary Figure 1. Workflow overview.

Supplementary Table 1. Elements of the myocarditis survey used in the current study

**Sociodemographic Information**	**Source of survey items**	**Data type**
1. Age (yrs) at survey initiation	Investigator-developed	Continuous
2. Sex at birth	Investigator-developed	Categorical [female, male, other]
3. Gender identity	Investigator-developed	Categorical [female, male, transgender man, transgender woman, genderqueer/non-conforming, other, no response/prefer not to say]
4. Race/ethnicity, self-reported	Investigator-developed	Categorical [White (non-Hispanic), Black, Latino/Hispanic American non-White, East Asian, South Asian, Middle Eastern/Arab, Native American/Alaskan Native, Other (specify), no response/prefer not to say]
5. Marital status	Investigator-developed	Categorical [Married/partnered, separated, widowed, divorced, other (specify), no response/prefer not to say]
6. Education (highest degree or level of school completed)	Investigator-developed	Categorical [no schooling, up to 12th grade with no diploma, high school diploma/general education degree, some college, associate's degree, bachelor's degree, master's degree, professional degree, doctorate degree, no response/prefer not to say]
7. Employment status	Investigator-developed	Categorical [working 40+ hours/week, working 1-39 hours/week, unemployed or laid off/looking for work, unemployed or laid off/not looking for work, keeping house or raising children full-time, retired, disabled/not able to work, other (specify), no response/prefer not to say]
8. Estimated annual income, household	Investigator-developed	Categorical [Less than $5K, $5K-$11,999, $12K-$15,999, $16K-$24,999, $25K-$34,999, $35K-$49,999, $50K-74,999, $75K-$99,999, $100K+, don't know, no response/prefer not to say]
9. Home location	Investigator-developed	Categorical [N. America, C. America, S. America, Europe, Africa, Asia, Australia, Caribbean Islands, Pacific Islands, other (specify), no response/prefer not to say
**Spirituality**	**Source of survey items**	**Data type**
10. Religious affiliation	Investigator-developed	Categorical, if religion is specified [Atheist/agnostic, Buddhism, Catholicism/Christianity, Hinduism, Islam, Judaism, other (specify), no response/prefer not to say]
11. Religiousness, self-reported	Investigator-developed	Categorical [Not religious, slightly religious, moderately religious, very religious, I don't know]
12. Spirituality, self-reported	Investigator-developed	Categorical [Not spiritual, slightly spiritual, moderately spiritual, very spiritual, I don't know]
**General Health**	**Source of survey items**	**Data type**

13. Perceived level of general health	Investigator-developed	Categorical [Excellent, very good, good, fair, poor]
14. Perceived level of physical fitness	Investigator-developed	Categorical [Excellent, very good, good, fair, poor]
15. Exercise level, weekly	Investigator-developed	Categorical [Daily, most days, a few days, only one to two days, none]
16. Sleep, hours/night	Investigator-developed	Continuous [Less than 3 hours…16+ hours]
17. Smoking status	Investigator-developed	Categorical [Never, former smoker, occasional smoker, daily smoker]
18. Frequency of alcohol use	Investigator-developed	Categorical [Not at all, less than once a month, 1-3 times a month, once weekly, 2-4 times per week, 5-6 times per week, at least once a day]
19. Drug use	Investigator-developed	Categorical [Never exposed, experimented without current use, current occasional use, current daily use, no response/prefer not to say]
**Personal and Family Mental Health History**	**Source of survey items**	**Data type**
20. Personal history of depression[a]	Investigator-developed	Categorical [No, yes-in the past but not current problem, yes-in the past and is a current problem, I don't know]
21. Personal history of anxiety[b]	Investigator-developed	Categorical [No, yes-in the past but not current problem, yes-in the past and is a current problem, I don't know]
22. Mental health disorders in primary relative(s)[c]	Investigator-developed	Categorical [No, yes (specify in other survey items), no response/prefer not to say]
23. Mental health disorders in secondary relative(s)[c]	Investigator-developed	Categorical [No, yes (specify in other survey items), no response/prefer not to say]
**Caregiving/Care receiving[d]**	**Source of survey items**	**Data type**
24. Caregiver status for activities of daily living (ADLs)	Investigator-developed	Categorical [Yes, no]
25. Caregiver status for instrumental ADLs	Investigator-developed	Categorical [Yes, no]
26. Receives assistance for instrumental ADLs	Investigator-developed	Categorical [Yes, no]
**Validated psychosocial assessments**	**Source of survey items**	**Data type**
27. Depressive Symptomatology	Center for Epidemiological Studies-Depression scale (CES-D)	Validated 20-item self-report measure with individual scale items ranging from 0 (rarely) to 3 most or all the time); clinically significant depressive symptoms defined as CES-D total score $\geq$16.
28. General anxiety	Beck Anxiety Inventory (BAI)	Validated 21-item self-report measure, with scale items ranging from 0 (not at all) to 3 (severe); clinically significant anxiety based on a validated cutoff of 22.
29. Traumatic distress	Revised Impact of Events Scale (IES-R)	Validated 22-item self-report measure with individual scale items ranging from 0 (not at all) to 4

		(extremely); clinically significant traumatic distress defined based on a validated cutoff of 24.
30. Health anxiety	Health Anxiety Inventory (HAI), short version	Validated 64-item self-report measure with individual items rated on a scale of 0 to 3 (higher
31. Physical quality of life and wellbeing	Linear Analog Self-Assessment (LASA), physical wellbeing subscale	Validated measure of overall and domain-specific quality of life and wellbeing, with each subscale rated on a scale of 0 (as bad as it can be) to 10 (as good as it can be).
32. Emotional quality of life and wellbeing	LASA, emotional wellbeing subscale	
33. Spiritual quality of life and wellbeing	LASA, spiritual wellbeing subscale	
34. Intellectual and mental quality of life and wellbeing	LASA, intellectual/mental wellbeing subscale	
35. Overall quality of life and wellbeing	LASA, overall wellbeing subscale	
36. Adverse childhood experiences	Adverse Childhood Experiences (ACE) questionnaire	Validated 10-item self-report measure with each item rated on a binary (yes/no) scale.
37. Treatment distress	Modification of the Cancer and Treatment Distress Scale (CTXD)[e]	Self-report measure developed for and validated in people with cancer, 17 specific items were adapted for use in this study, each rated on a scale of 0 (none) to 3 (severe).
38. Social support	ENRICHD Social Support Instrument (ESSI)	7-item self-report measure rated on a scale ranging from 1 (none of the time) to 5 (all of the time). Item 7 (marital status) is rated as 4 (yes) or 2 (no).
39. Resiliency	Brief Resilience Scale (BRS)	6-item self-report measure with individual scale items rated on a 5-point Likert scale ranging from 1 ([or 5 for reverse-coded items] strongly disagree) to 5 ([or 1 for reverse-coded items] strongly agree).
**Myocarditis-specific variables and somatic symptom burden**	**Source of survey items**	**Data type**
40. Age at myocarditis diagnosis	Investigator-developed	Continuous
41. Myocarditis cause	Investigator-developed	Categorical [Viral, bacterial, another type of infection, Giant cell, immune disease (specify), thyroid disease, allergic reaction to medicine (specify), toxic effect of medicine, alcohol or drugs (specify), other cause (specify), unknown cause]
42. Estimated survival time since diagnosis	Calculated	Calculated using age at the date of survey initiation and approximate age at myocarditis diagnosis
43. Use of an AICD since myocarditis diagnosis	Investigator-developed	Categorical [Yes, no]
44. Use of a device to maintain cardiac function since myocarditis diagnosis[f]	Investigator-developed	Categorical [Yes, no]
45. Myocardial transplant status	Investigator-developed	Categorical [Yes, no]

46. Number of symptoms potentially ascribable to cardiac cause (number of cardiac symptoms)	15-item Public Health Questionnaire (PHQ-15)	The PHQ-15 is a 15-item self-report measure with individual items ranging from 0 (not bothered at all) to 2 (bothered a lot). Individual items from the full 15-item scale representing symptoms potentially ascribable to a cardiac cause included items 6 (chest pain), 7 (dizziness), 8 (fainting spells), 9 (feeling your heart pound or race), 10 (shortness of breath), and 14 (feeling tired or having low energy). For each of the 6 cardiac symptoms, respondents were asked to rate perceived changes in severity over the preceding 6 months (or since myocarditis diagnosis if <6 months) on the following scale: 0 (no change), 1 (a little worse), and 2 (a lot worse).
47. Perceived burden from symptoms potentially ascribable to cardiac cause (cardiac symptom burden)	PHQ-15	
48. Somatic symptom burden	PHQ-15	

[a] Sub-items also assessed for specific diagnoses (major depression, persistent depressive disorder, or other depression), timing of depression symptoms relative to the estimated date of myocarditis diagnosis, perceived likelihood that depression is related specifically to having myocarditis, and depression treatment history. [b] Sub-items also assessed for specific diagnoses (generalized anxiety disorder, panic attacks, phobias [including social phobia or social anxiety disorder], post-traumatic stress disorder), timing of anxiety symptoms relative to the estimated date of myocarditis diagnosis, perceived likelihood that anxiety is related specifically to having myocarditis, and anxiety treatment history. [c] Sub-items assessed the number of primary (or secondary) relatives with at least one diagnosed mental health condition and specific conditions that were diagnosed. [d] Caregiving and care receiving items assessed whether respondents were providing caregiving to others for activities of daily living (ADLs) or instrumental ADLs or were receiving caregiving from others for instrumental ADLs. [e] The modified CTXD used in this study retained items assessing distress related to inability to do things respondents used to do, long-term effects of treatment, dealing with the medical system, wondering about emotional toll on family or caregivers, changes in appearance, dealing with insurance, thinking about things that could go wrong, inability to take care of family, potential problems supporting the family financially, not having the same family responsibilities, returning to work, communicating with healthcare workers, getting information, possible death, costs of care, needing help from others, and feeling like a burden to others. [f] Including use of a ventricular assist device, intra-aortic balloon pump, or extracorporeal membrane oxygenation.

Supplementary Table 2. List of variables used in machine learning methods for predicting clinically significant depressive symptomatology.

**Depressive Symptomatology Variables**	
1	Age (yrs) at survey initiation
2	Sex at birth
3	Self-described gender identity
4	Self-described race
5	Self-described marital status
6	Highest degree earned
7	Current employment status
8	Estimated annual income (during the last 12 mo), USD
9	Geographical home location
10	Self-reported religion
11	Self-reported level of religiousness
12	Self-reported spirituality
13	Self-reported level of general health
14	Self-reported level of fitness
15	Self-reported exercise level
16	Self-reported average number of hrs of sleep per night in past month
17	Smoking status
18	Frequency of alcohol use
19	Self-reported drug use
20	History of depression
21	History of anxiety
22	Mental health disorders, primary relatives
23	Mental health disorders, secondary relatives
24	Caregiver status

25	Assistance given for IADLs
26	Assistance received for IADLs
27	BAI Total Score
28	IES-R Total Score
29	HAI-18 Total Score
30	LASA1: Overall Physical Wellbeing
31	LASA2: Overall Emotional Wellbeing
32	LASA3: Overall Spiritual Wellbeing
33	LASA4: Overall Intellectual/Mental Wellbeing
34	LASA5: Overall Quality of Life
35	ESSI Total Score
36	ACE Total Score
37	Modified CTXD Total Score
38	Cause of myocarditis
39	Estimated survival since diagnosis
40	AICD use
41	Device needed to maintain cardiac fx
42	Myocardial transplant status
43	Number of PHQ-15 cardiac symptoms
44	Sum of PHQ-15 items measuring cardiac symptoms
45	Average of perceived cardiac symptoms scores
46	BRS Total Score

Supplementary Table 3. Scores on psychosocial measures and measures of general somatic and cardiac symptom burden, overall and in depressed (clinically significant depressive symptomatology) and non-depressed respondents

	Overall cohort	Depressed	Non-depressed	p-value
	n = 96	n = 43	n = 53	
	Median (IQR)	Median (IQR)	Median (IQR)	
CES-D total score	13.0 (6.8, 27.2)	29.0 (22.2, 36.0)	7.0 (4.0-10.0)	<0.001
BAI total score	11.0 (5.0-21.0)	18.0 (12.0-26.5)	5.0 (3.0-10.8)	<0.001
HAI total score	18.0 (13.0-25.0)	23.0 (17.0-33.0)	14.0 (10.0-20.0)	<0.001
IES-R total score	16.0 (3.0-30.5)	29.0 (16.8-48.0)	7.0 (1.8-16.2)	<0.001
ESSI total score	30.0 (26.0-33.0)	27.0 (19.5-31.0)	31.0 (29.0-33.0)	<0.001
BRS total score	21.0 (16.2-24.0)	18.0 (14.0-20.0)	24.0 (20.0-25.2)	<0.001
LASA, overall	7.0 (4.0-8.0)	4.5 (3.0-6.0)	8.0 (7.0-9.0)	<0.001
ACE total score	1.0 (0.0-3.0)	2.0 (1.0-3.8)	1.0 (0.0-2.0)	<0.001
Modified CTXD	14.0 (6.0-24.5)	23.0 (11.0-30.8)	11.0 (4.0-18.0)	<0.001
PHQ-15 total score	9.0 (5.4-12.7)	11.0 (7.8-14.3)	5.0 (2.0-8.0)	<0.001
Number of cardiac symptoms[a]	3.0 (1.0-4.0)	3.0 (2.0-5.0)	2.0 (1.0-3.3)	0.002
Cardiac symptom severity[b]	2.0 (0.0-4.0)	3.0 (1.3-4.8)	1.0 (0.0-3.0)	0.007

Key: ACE = Adverse Childhood Experiences questionnaire; BAI = Beck Anxiety Inventory; BRS = Brief Resilience Scale; CES-D = Center for Epidemiologic Studies Depression Scale; ESSI = ENRICHD Social Support Instrument; HAI = Health Anxiety Inventory (short form); IES-R = revised Impact of Events Scale; LASA = Linear Analog Self-Assessment scale; Modified CTXD = modified version of the Cancer and Treatment Distress Scale; PHQ-15 = 15-item Public Health Questionnaire.

[a] Assessed using items 6 (chest pain), 7 (dizziness), 8 (fainting spells), 9 (feeling your heart pound or race), 10 (shortness of breath), and 14 (feeling tired or having low energy) of the 15-item Public Health Questionnaire (PHQ-15). [b] Defined as the average symptom severity rating for PHQ-15 items 6, 7, 8, 9, 10, and 14.

Supplementary Table 4. Variable importance (VI) of variables used in random forests models predicting clinically significant depressive symptomatology

Random Forest – Clinically Significant Depressive Symptomatology			
	Variable	Mean VI	SD VI
1	LASA emotional subscale	87.87	23.9
2	LASA overall	76.08	25.59
3	History of depression	71.86	23.44
4	BAI total	66.83	20.48
5	BRS total	54.35	19.5
6	IES-R total	53.78	22.02
7	LASA intellectual/mental subscale	53.05	23.72
8	History of anxiety	38.82	20.99
9	LASA spiritual subscale	35.17	18.04
10	Religious affiliation	31.3	22.49
11	LASA physical subscale	29.58	14.11
12	Religiousness	27.21	14.8
13	HAI total	26.95	13.99
14	ESSI total	25.95	20.15
15	Modified CTXD total	24.94	18.37
16	ACE total	24.45	16.31
17	Employment status, current	23.92	10.05
18	Drug use	21.43	15.5
19	Age	19.9	11.79
20	Exercise level	19.43	12.18
21	Smoking status	16.55	9.37
22	Family MH history, primary relative	14.87	11.41
23	Average perception score on cardiac functioning	14.7	9.74
24	No. of cardiac symptoms	12.47	8.41
25	Perceived general health	11.13	10.49
26	Spirituality	10.01	6.87
27	Estimated survival since diagnosis	8.9	8.11
28	Frequency of alcohol use	6.38	7.18
29	Cardiac symptom score	5.79	9.08
30	Fitness level, perceived	5.68	11.12
31	Marital status	5.64	7.4
32	Family MH history, secondary relative	4.93	3.91
33	Sex	4.4	4.87
34	Gender identity	3.74	4.92
35	Device needed to maintain cardiac function	2.66	6.39

36	Geographical home location	1.94	2.45
37	Assistance received for IADLs	1.75	3.78
38	Annual income	0.93	4.7
39	Caregiver status	0.84	4.32
40	Hours of sleep, average	0.55	7.5
41	Race	0.53	1.68
42	Myocardial transplant status	0.33	2.71
43	Cause of myocarditis	-0.12	4.91
44	Highest degree earned	-0.46	3.92
45	AICD use	-1.27	3.22

Supplementary Table 5. Variable importance (VI) of variables used in XGBoost models predicting clinically significant depressive symptomatology

XGBoost – Clinically Significant Depressive symptomatology			
	Variable	Mean VI	SD VI
1	LASA emotional subscale	69.65	32.29
2	BAI total	60.56	37.68
3	IES-R total	58.33	38.81
4	History of depression	47.01	35.39
5	LASA overall	43.85	28.2
6	LASA intellectual/mental subscale	43.57	33.32
7	History of anxiety	35.48	29.12
8	BRS total	30.29	31.14
9	ESSI total	23.71	30.88
10	ACE total	20.78	27.73
11	LASA physical subscale	18.01	28.46
12	HAI total	16.18	17.32
13	Employment status, current	13.19	17.98
14	Age	9.02	9.74
15	Religious affiliation	8.89	13.55
16	Modified CTXD total	7.84	7.88
17	LASA spiritual subscale	7.39	8.81
18	Highest degree earned	7.23	9.69
19	Family MH history, primary relative	5.46	6.6
20	Average perception score on cardiac functioning	4.73	7.97
21	Estimated survival since diagnosis	4.66	2.89
22	Exercise level	4.27	2.14
23	Frequency of alcohol use	2.86	4.24
24	Drug use	2.65	3.39
25	Religiousness	2.54	3.14
26	Annual income	2.32	2.86
27	Perceived general health	2.16	1.68
28	No. of cardiac symptoms	2.14	1.54
29	Cause of myocarditis	1.93	1.97
30	Race	1.86	2.07
31	Cardiac symptom score	1.76	1.52
32	Fitness level, perceived	1.3	0.78
33	Spirituality	1.22	1.33
34	Marital Status	0.86	0.6
35	Hours of sleep, average	0.78	0.55

36	Family MH history, secondary relative	0.68	0.62
37	Smoking status	0.65	0.72
38	Gender identity	0.23	0.48
39	Sex	0.21	0.31
40	Caregiver status	0.13	0.03
41	AICD use	0.07	0.03
42	Device needed to maintain cardiac function	0.05	NA

Supplementary Table 6. Variable importance (VI) of variables used in penalized regression models predicting clinically significant depressive symptomatology

Penalized Regression – Clinically Significant Depressive Symptomatology			
	Variable	Mean VI	SD VI
1	Smoking status	78.47	24.3
2	Caregiver status	61.68	29.33
3	Employment status, current	61.13	23.14
4	BAI total	59.05	25.45
5	LASA overall	49.66	18.91
6	BRS total	43.83	29.47
7	Cause of myocarditis	42.21	29.05
8	Race	38.76	27.36
9	Annual income	38.03	31.95
10	Religious affiliation	37.94	30.96
11	IES-R total	37.82	28.97
12	LASA emotional subscale	36.55	14.41
13	Assistance received for IADLs	35.14	32.26
14	Average perception score on cardiac functioning	34.84	23.04
15	LASA intellectual/mental subscale	33.31	19.22
16	Highest degree earned	33.05	30.42
17	History of depression	30.72	19.15
18	Cardiac symptom score	28.69	18.02
19	ESSI total	28.21	24.89
20	Fitness level, perceived	27.49	15.32
21	Device needed to maintain cardiac function	27.13	23.11
22	ACE total	26.63	22.3
23	HAI total	25.61	15.37
24	History of anxiety	22.36	20.11
25	Perceived general health	21.9	19.76
26	Modified CTXD total	21.34	28.09
27	Age	20.88	17.64
28	Drug use	20.68	25.36
29	No. of cardiac symptoms	19.47	16.48
30	Exercise level	19.2	17.09
31	LASA spiritual subscale	18.86	20.83
32	Sex	16.24	16.75
33	Assistance given for IADLs	15.26	13.53
34	Family MH history, secondary relative	14.55	9.11
35	AICD use	14.23	13.84

36	Family MH history, primary relative	13.32	14.27
37	Frequency of alcohol use	13.22	16.25
38	Myocardial transplant status	13.06	16.84
39	Religiousness	12.66	12.89
40	Hours of sleep, average	10.98	12.43
41	Estimated survival since diagnosis	9.54	5.95
42	Marital status	9.28	9.19
43	Spirituality	9.16	15.57
44	LASA physical subscale	7.05	9.74
45	Geographical home location	4.5	6.6
46	Gender identity	3.74	4.81

Supplementary Table 7. Results of logistic regression analyses examining the independent associations of clinically significant depressive symptomatology with top predictors from Random Forest (best performing) predictive models,

Depressive Symptomatology Logistic Regression		
Predictor	OR[a]	95% CI
Emotional well-being[b]	0.34	0.19, 0.59
Overall quality of life[c]	0.45	0.30, 0.67
Resilience[d]	0.82	0.70, 0.96
Traumatic distress[e]	1.09	1.04, 1.15
Anxiety, general[f]	1.12	1.04, 1.21
[a] Logistic regression models were adjusted for age at survey initiation, sex at birth (self-reported), smoking status, alcohol intake, and self-reported history of depression. [b] Assessed using the LASA emotional wellbeing subscale. [c] Assessed using the Linear Analog Self-Assessment (LASA) overall wellbeing subscale. [d] Assessed using the Brief Resilience Scale (BRS). [e] Assessed using the revised Impact of Events Scale (IES-R). [f] Assessed using the Beck Anxiety Inventory (BAI).		
